# DNA methylation associated with postpartum depressive symptoms overlaps findings from a genome-wide association meta-analysis of depression

**DOI:** 10.1186/s13148-019-0769-z

**Published:** 2019-11-28

**Authors:** Dana M. Lapato, Roxann Roberson-Nay, Robert M. Kirkpatrick, Bradley T. Webb, Timothy P. York, Patricia A. Kinser

**Affiliations:** 1 Department of Human and Molecular Genetics, Virginia Commonwealth University, Richmond, VA USA; 2 Virginia Institute for Psychiatric and Behavioral Genetics, Virginia Commonwealth University, Richmond, VA USA; 3 Department of Psychiatry, Virginia Commonwealth University, Richmond, VA USA; 4 Department of Obstetrics and Gynecology, Virginia Commonwealth University, Richmond, VA USA; 5 School of Nursing, Virginia Commonwealth University, Richmond, VA USA

**Keywords:** DNA methylation, Perinatal depressive psychopathology, Epigenome-wide association study, Differentially methylated regions, Major depression

## Abstract

**Background:**

Perinatal depressive symptoms have been linked to adverse maternal and infant health outcomes. The etiology associated with perinatal depressive psychopathology is poorly understood, but accumulating evidence suggests that understanding inter-individual differences in DNA methylation (DNAm) patterning may provide insight regarding the genomic regions salient to the risk liability of perinatal depressive psychopathology.

**Results:**

Genome-wide DNAm was measured in maternal peripheral blood using the Infinium MethylationEPIC microarray. Ninety-two participants (46% African-American) had DNAm samples that passed all quality control metrics, and all participants were within 7 months of delivery. Linear models were constructed to identify differentially methylated sites and regions, and permutation testing was utilized to assess significance. Differentially methylated regions (DMRs) were defined as genomic regions of consistent DNAm change with at least two probes within 1 kb of each other. Maternal age, current smoking status, estimated cell-type proportions, ancestry-relevant principal components, days since delivery, and chip position served as covariates to adjust for technical and biological factors. Current postpartum depressive symptoms were measured using the Edinburgh Postnatal Depression Scale. Ninety-eight DMRs were significant (false discovery rate < 5%) and overlapped 92 genes. Three of the regions overlap loci from the latest Psychiatric Genomics Consortium meta-analysis of depression.

**Conclusions:**

Many of the genes identified in this analysis corroborate previous allelic, transcriptomic, and DNAm association results related to depressive phenotypes. Future work should integrate data from multi-omic platforms to understand the functional relevance of these DMRs and refine DNAm association results by limiting phenotypic heterogeneity and clarifying if DNAm differences relate to the timing of onset, severity, duration of perinatal mental health outcomes of the current pregnancy or to previous history of depressive psychopathology.

## Introduction

Perinatal depressive symptoms can occur any time during pregnancy or shortly following birth and even subclinical levels of depressive symptoms have been associated with an increased risk for episodes of major depression with onset in the peripartum (MDP), pregnancy complications, maternal suicide, and adverse infant health outcomes and development [1–7]. The Diagnostic and Statistical Manual (fifth edition; DSM-5) classifies MDP as a major depressive episode that occurs during pregnancy or within 4 weeks of delivery [8]; however, in practice, researchers and clinicians may extend this period to up to one year postpartum. Attempts to understand the impact of depressive psychopathology on biological mechanisms have primarily centered on elucidating the relationship between maternal mental health and negative infant outcomes. As a result, relatively little is known regarding how perinatal depressive symptoms affect maternal biological processes. Epidemiological studies suggest that episodes of major depression (MD) may increase the risk for other adverse health outcomes, such as cardiovascular disease, and perturb immune system activities. The biological pathways associated with these persistent changes in immune activity and elevated risk have not yet been identified. More work is needed to uncover the pathways underlying these comorbidities and to determine if the biological impact from depressive symptoms differs by clinical subtype (e.g., perinatal and early-onset). One potential avenue for understanding biological changes associated with depressive psychopathology is through DNA methylation (DNAm) studies.

DNAm is a chemical modification typically found on cytosines bordering guanines (i.e., cytosine-phosphate-guanine [CpG] sites). DNAm can influence gene expression, genomic stability, and chromatin conformation [9]. Inter-individual differences in DNAm have been associated with early mortality [10], cancer [11], imprinting disorders [12, 13], childhood trauma exposure [14], biological age [15, 16], and schizophrenia [17, 18]. Associations between DNAm and clinical MD and/or depressive symptoms proximal to [19–22] or absent pregnancy [23–26] have been reported; however, most of the perinatal depression studies have focused on identifying DNAm patterns in fetal tissues associated with maternal mental health, leaving a significant knowledge gap. Characterizing the relationship between DNAm and perinatal depressive symptoms may provide insight regarding the pathoetiology of MDP as well as potentially identify biological markers [27].

This study sought to identify DNAm patterns associated with perinatal depressive symptoms during the first 7 months postpartum in maternal blood focusing on regional DNAm changes. Genome-wide DNAm and repeated measures of maternal mental health were collected as part of a longitudinal study of preterm birth [28]. The rationale for focusing on differentially methylated regions (DMRs) rather than single CpG site associations was twofold. One, regional changes are thought to represent differences more likely to be biologically meaningful and statistically credible [29]. A single CpG site associated with a trait could be spurious; however, multiple CpG sites within one region each associating with a trait in the same direction is likely to represent a more robust finding. Two, regional analyses reduce the burden of multiple tests and allow one to test for smaller probe effect sizes [30]. The sample size for this study is acceptable to test regional differences, but it is not well-powered to identify individual probe associations.

## Methods

### Study participants

The data for this analysis come from the Pregnancy, Race, Environment, Genes (PREG) study and its postpartum extension [28]. Both PREG and the extension received IRB approval, and all participants provided written informed consent for both parts. Most participants had the first postpartum visit within 3 months of delivery and the second visit within 9 months. The PREG study recruited an epidemiological sample of women in early pregnancy primarily from two health clinics in Richmond, Virginia. The purpose of the PREG study was to identify factors that influence racial health disparities in preterm birth. The postpartum study extension permitted additional perinatal outcomes, like perinatal depression, to be investigated.

### Study eligibility criteria

PREG study enrollment criteria required participants to (1) be < 24 weeks gestation, (2) have a singleton pregnancy, (3) have not used artificial reproductive technology for the current pregnancy, (4) be between 18 and 40 years old, and (5) be absent of major health conditions (e.g., diabetes) [28]. Additionally, the participant and the biological father had to self-identify as either both African-American or both European-American and without Middle Eastern or Hispanic ancestry. Birth exclusion criteria included chromosomal or amniotic abnormalities (e.g., polyhydramnios/oligohydramnios) and congenital birth defects.

### Psychiatric assessments

Current perinatal depressive symptoms were measured at both postpartum study visits using the Edinburgh Postnatal Depression Scale (EPDS) [31]. The EPDS is a 10-item self-report instrument that is used in clinical practice to screen for probable perinatal depression and in research to assess for perinatal depressive symptoms; it differs from other depressive symptom measures because the items focus on symptoms specifically related to depression that would not be part of a typical pregnancy [32]. For example, difficulty sleeping is common in pregnancies regardless of maternal MD status. EPDS total score was analyzed as a continuous variable. Scores of 13 or more out of 30 indicate probable perinatal depression [31].

### Genome-wide DNAm measurement and processing

Maternal genome-wide DNAm was assayed according to the manufacturer’s protocol (Illumina, San Diego, CA, USA) from peripheral blood using the EPIC beadchip, which includes more than 850,000 probes and interrogates regulatory, genic, and intergenic regions [33]. Blood samples were collected in EDTA tubes at each study visit along with health questionnaires. An aliquot of 1 μg DNA per participant was sent to HudsonAlpha Laboratories for DNAm measurement. Samples were randomized to arrays to minimize potential batch effects related to processing influencing DNAm patterns. Peripheral blood was selected given its accessibility, the availability of cell-type deconvolution methods, and the strong evidence of immune system involvement in MDP pathophysiology [34–37].

Raw microarray data was processed using Bioconductor packages in the R environment in line with best practices [38–40] (version 3.5). Signal intensity and probe failure rate were evaluated using the *minfi* [41] package to identify poor quality samples and probes. Samples were removed if either the median unmethylated or methylated signal intensities were less than 10.5. Probes were removed if they failed in > 1% of samples (*n* = 12,557), overlapped single-nucleotide polymorphisms (SNPs; *n* = 30,435), or had been identified as cross-hybridizing in the Illumina Infinium HumanMethylation450 beadchip (predecessor technology) [42]. Probes on the sex chromosomes were retained given that the entire sample was female, leaving a total of 782,884 probes. All samples were quantile normalized, and blood cell type proportions were estimated using the Houseman method [43]. Sample identity was confirmed using the 59 control probes on the EPIC beadchip. These probes overlap polymorphic sites and in aggregate can estimate sample relatedness and detect sample duplication. All pairwise sample correlations were calculated. Any sample correlated too poorly with its sister samples (*r* < 0.8) or too highly with samples from another person (*r* > 0.6) were removed or relabeled if the correct identity could be ascertained. Only one blood sample per person was used for this analysis. In general, DNAm samples from the first postpartum visit were used; however, if a participant’s first postpartum visit failed quality control and she had a second postpartum DNAm sample within 7 months of delivery, then that sample and the EPDS questionnaire from the same visit was used. For all participants, the phenotypic and DNAm data were from the same study visit. The purpose for using only one sample was to capture the postpartum time period with the highest estimates of depressive symptom prevalence while also limiting potential phenotypic heterogeneity [44–46].

## DNAm analysis

### Covariate selection

Maternal age, number of days postpartum at blood and questionnaire collection, smoking status, and microarray row were selected as covariates a priori based on known or putative associations with DNAm. Principal component analysis (PCA) was applied to the normalized methyl values [47] of the filtered probe set, and correlations between the top ten principal components (PC) and technical and biological variables were plotted to identify additional potential confounders. Four variables (estimated granulocyte proportion, chip size, self-reported census-based race category, and slide ID number) correlated greater than the absolute value of 0.5 with at least one PC. Slide effects were addressed using ComBat [48]. Genetic ancestry relevant PCs were calculated with the Barfield method [49]. Two PCs strongly independently correlated with self-identified race were included instead of self-identified race because the PCs have been shown to adjust for genomic inflation better than a categorical variable [49]. These two components also correlated strongly with estimated blood cell proportions (*r* > than the absolute value of 0.5). Estimated granulocyte proportion also was included as a covariate to provide additional adjustment for cell-type heterogeneity.

### Identifying single-site and regional DNAm changes

The methyl values for individual probes were regressed onto postpartum EPDS total score using the *limma* package [50]. Covariates controlling for row, granulocyte proportion, median unmethylated signal intensity maternal age, gestational age at blood collection, smoking status, and allelic ancestry were included. The strength of the association between individual probes and EPDS total score was evaluated using empirical *p* values derived from a null distribution derived from the dataset itself (*k* = 20,000 permutations). This analysis strategy provides a more appropriate assessment and adjustment for test statistic inflation than a metric like lambda [51]. The median effect size for probes used in the DMR analysis was assessed using the difference in adjusted *R*^2^ values between the full model and a reduced model without EPDS total score.

The probes with the largest observed *t* statistics (top and bottom 2.5% of tested probes) were used for regional analysis. Differentially methylated regions (DMRs) were defined as contiguous regions of consistent DNAm change (i.e., all hypermethylated or all hypomethylated) that contained at least two probes within 1 kb using a method similar to that described by Ong and Holbrook [30]. This strategy is similar to the DMRcate algorithm in that only the subset of the probes with the best evidence for association is used to construct DMRs [52].

DMR significance was assessed using a rank-based permutation strategy and an empirical null distribution derived from *k* = 1000 permutations. DMRs were constructed from both the observed data and 1000 of the 20,000 DMP permutation sets. For each DMR, the area under the curve (AUC) was calculated using the trapezoidal rule, where each probe’s *t* statistic served as height and the distance between the probes as width. Thus, the magnitude of the AUC reflects both the strength of each probe’s association (height) and the size of the region (width). The significance of analysis microarray (SAM) method was implemented to assign test statistics to each observed DMR [53]. This method ranks all DMRs generated within a permutation by AUC and performs row-wise comparisons between the ranked observed DMRs and the ranked permutation DMRs to calculate a false discovery rate (FDR).

### Gene set enrichment and comparison to other genetic findings related to depression

DMPs and DMRs were annotated using AnnotationHub [54]. Gene set enrichment testing for functional and regulatory roles was performed on the combined dataset of DMPs and DMRs using Entrez IDs and clusterProfiler [55]. The rationale for combining DMRs and DMPs into a single group for gene set analysis was to address the issue that not all probes are capable of forming DMRs. In order to give those regions of the genome an opportunity to contribute to gene set enrichment analysis, DMPs and DMRs were analyzed together (see Additional file 1 for full enrichment analysis of the combined DMR-DMP analysis as well as the DMP-only and DMR-only enrichment analyses performed separately with clusterProfiler [55] and methylGSA [56]).

The results from this analysis were compared directly to two studies of depression: the latest Psychiatric Genomics Consortium (PGC) meta-analysis of genome-wide association studies of depression and an epigenome-wide association study (EWAS) of early-onset MD. For the PGC study, the 44 significant loci were obtained to determine the extent of overlap with significant DNAm regions [57]. Bootstrap and permutation methods (*k* = 1000) were used to test if DNAm regions were enriched for PGC loci. For the early-onset MD EWAS, site, regional, and gene enrichment results from the Adolescent and Young Adult Twin Study (AYATS) were compared to findings from this study to determine the extent of overlap and similarity [26, 85].

## Results

### Sample characteristics

Sample demographics can be found in Table 1 and are representative of Richmond, Virginia. Approximately half of the participants (46%) self-identified as African-American, and the mean gestational age was 274.5 days (sd = 13.0 days). Most of the women (65%) were primigravida and had full-term pregnancies (94%). Very few participants were current smokers (8%), and 18% of the total sample self-reported a positive lifetime history of MD. Lifetime MD history did not significantly predict postpartum EPDS score, and the distribution of EPDS scores by self-reported MD history are in Additional file 5: Figure S3. The average EPDS score was 5.4, and 13 participants (14%) scored 13 or greater on the EPDS. The average time between delivery and postpartum study visit was 57 days (see Additional file 5).

**Table 1:** PREG-PPD sample characteristics

Demographics	
Total *N*	92
African-American (%)	42 (45.7)
Age	29.7 (4.4)
Early gestation BMI	27.4 (7.3)
First pregnancy (%)	59 (64.8)
Gestational length (days)	274.5 (13.0)
Preterm birth (%)	6 (6.5)
Days postpartum^a^	56.5 (36.7)
Current smoker (%)	7 (7.6)
Depressive phenotypes	
Positive lifetime history of MD (%)^b^	16 (18.0)
Current postpartum depressive symptom load^c^	5.4 (5.9)

*PREG-PPD* Pregnancy, Race, Environment, Genes study Postpartum Extension; *MD* major depression

*All values are mean (standard deviation) unless otherwise noted with '(%)' to indicate N (%) or 'N' to indicate count. Percentages were calculated using valid responses

^a^Number of days postpartum when study visit (i.e., blood draw and questionnaire collection) occurred

^b^Assessed using the Composite International Diagnostic Interview-Short Form

^c^Assessed using the Edinburgh Postnatal Depression Scale

### Differentially methylated probes and regions

After microarray quality control and processing, 782,884 probes remained for analysis. From this filtered probe set, it was possible to create up to 109,340 background DMRs. *N* = 206,804 probes were ineligible to participate in regional analyses because they did not have a neighboring probe within 1 kb. Individual site analysis of the entire probe set identified 50 DMPs significantly associated with EPDS total score (empirical *p* = 0 after 20,000 permutations; see Additional file 5 for DMP quantile-quantile plot). Approximately 39,150 probes were taken forward for regional analysis. The median difference in adjusted *R*^2^ values for the full versus reduced models for these probes was 0.053 (interquartile range = 0.02). Ninety-eight genomic regions spanning a collective 116.2 kb were significantly differentially methylated by postpartum depressive symptom load (FDR < 5%). The significant regions overlapped 92 genes on 20 chromosomes (none on chromosomes 7 or 18), and on average spanned ~ 1.2 kb in length (see Additional file 2 for full DMR gene list). The number of CpG probes in significant DMRs ranged from 2 to 10 (mean = 3.48 probes).

### Gene set enrichment and comparison of DNAm patterns associated with postpartum depressive symptoms and other genetic findings related to depression

Detailed results for the combined gene set enrichment of DMPs and DMRs can be found in Table 2. In short, the combined analysis identified one biological process (BP; cognition) and four cellular components (CC; DNA repair complex, neuron to neuron synapse, axon part, and synapse part) significant at an FDR < 5%. The DMP and DMR only analyses did not identify any categories with an FDR < 5% and produced dissimilar results (see Additional file 1). The DMP only analysis identified multiple BP and CC categories associated with neural processes (e.g., central nervous system neuron development, transmission of nerve impulses, somatodendritic compartment, and neuronal cell body), and the significance of these categories was markedly attenuated using a gene enrichment algorithm that adjusts for number of probes per gene [56]. The DMR only analysis returned Gene Ontology (GO) categories from a variety of biological systems, including platelet formation and morphogenesis, cardiac muscle tissue development, chemical synaptic transmission, inflammatory cell apoptotic process, and tissue morphogenesis.

**Table 2:** Gene Ontology results for differentially methylated probes and regions

Ontology	Description	Gene ratio	Bg ratio	*p* value	*q* value	Gene ID
BP	Cognition	11/136	284/17397	0.0000	0.038	TTC8/ADORA1/CNTNAP2/MEF2C/MEIS2/NTSR1/PAFAH1B1/ADGRB3/RASGRF1/SLC6A4/SYNGAP1
BP	Learning or memory	9/136	246/17397	0.0001	0.114	CNTNAP2/MEF2C/MEIS2/NTSR1/PAFAH1B1/ADGRB3/RASGRF1/SLC6A4/SYNGAP1
BP	Detection of temperature stimulus involved in sensory perception	3/136	15/17397	0.0002	0.114	ADORA1/ARRB2/NTSR1
BP	Detection of temperature stimulus involved in sensory perception of pain	3/136	15/17397	0.0002	0.114	ADORA1/ARRB2/NTSR1
BP	Dendrite development	8/136	216/17397	0.0003	0.114	CTNND2/COBL/MAP2/MEF2C/PAFAH1B1/ADGRB3/KLF7/SYNGAP1
BP	Detection of temperature stimulus	3/136	19/17397	0.0004	0.114	ADORA1/ARRB2/NTSR1
BP	Modulation of chemical synaptic transmission	11/136	417/17397	0.0004	0.114	ADORA1/SYT9/ARRB2/MEF2C/NTSR1/RASGRF1/SLC6A4/TMEM108/YWHAG/SYNGAP1/CLSTN3
BP	Regulation of trans-synaptic signal	11/136	418/17397	0.0004	0.114	ADORA1/SYT9/ARRB2/MEF2C/NTSR1/RASGRF1/SLC6A4/TMEM108/YWHAG/SYNGAP1/CLSTN3
BP	Platelet formation	3/136	20/17397	0.0005	0.114	ZFPM1/MEF2C/MYH9
BP	Establishment of cell polarity	6/136	128/17397	0.0005	0.114	SDCCAG8/SH3BP1/MAP2/MYH9/PAFAH1B1/FRMD4A
BP	Actin filament-based process	15/136	723/17397	0.0005	0.114	ABI2/ADORA1/DIAPH2/COBL/SH3BP1/KCNJ5/MEF2C/MYH9/NEB/PAFAH1B1/PLS3/LURAP1/ACTN4/MYOM2/TBCK
BP	Platelet morphogenesis	3/136	21/17397	0.0006	0.114	ZFPM1/MEF2C/MYH9
BP	Sensory perception of temperature stimulus	3/136	21/17397	0.0006	0.114	ADORA1/ARRB2/NTSR1
BP	Behavior	13/136	579/17397	0.0006	0.116	ADORA1/CNTNAP2/KCND2/ARRB2/MEF2C/MEIS2/NTSR1/PAFAH1B1/PEX13/ADGRB3/RASGRF1/SLC6A4/SYNGAP1
BP	Response to hypoxia	9/136	308/17397	0.0007	0.125	ADORA1/HILPDA/KCND2/LMNA/MMP2/HIF3A/SLC6A4/TGFBR2/ACTN4
BP	Regulation of postsynaptic membrane	6/136	139/17397	0.0008	0.128	ADORA1/KCND2/ARRB2/MEF2C/NTSR1/TMEM108
BP	Response to decreased oxygen levels	9/136	319/17397	0.0009	0.128	ADORA1/HILPDA/KCND2/LMNA/MMP2/HIF3A/SLC6A4/TGFBR2/ACTN4
BP	Chemical synaptic transmission	14/136	685/17397	0.0010	0.128	ADORA1/SYT9/GAD2/KCND2/ARRB2/MEF2C/NTSR1/PAFAH1B1/RASGRF1/SLC6A4/TMEM108/YWHAG/SYNGAP1/CLSTN3
BP	Anterograde trans-synaptic signaling	14/136	685/17397	0.0010	0.128	ADORA1/SYT9/GAD2/KCND2/ARRB2/MEF2C/NTSR1/PAFAH1B1/RASGRF1/SLC6A4/TMEM108/YWHAG/SYNGAP1/CLSTN3
BP	Establishment or maintenance of cell polarity	7/136	199/17397	0.0010	0.128	SDCCAG8/SH3BP1/LMNA/MAP2/MYH9/PAFAH1B1/FRMD4A
BP	Trans-synaptic signaling	14/136	693/17397	0.0011	0.134	ADORA1/SYT9/GAD2/KCND2/ARRB2/MEF2C/NTSR1/PAFAH1B1/RASGRF1/SLC6A4/TMEM108/YWHAG/SYNGAP1/CLSTN3
BP	Synaptic signaling	14/136	698/17397	0.0011	0.137	ADORA1/SYT9/GAD2/KCND2/ARRB2/MEF2C/NTSR1/PAFAH1B1/RASGRF1/SLC6A4/TMEM108/YWHAG/SYNGAP1/CLSTN3
CC	DNA repair complex	4/141	42/18363	0.0003	0.044	CETN3/ERCC1/PAXX/WRN
CC	Neuron to neuron synapse	10/141	340/18363	0.0003	0.044	ADORA1/SYT9/CTNND2/KCND2/ARRB2/MAP2/NTSR1/TMEM108/SYNGAP1/CLSTN3
CC	Axon part	10/141	373/18363	0.0006	0.049	ADORA1/COBL/CNTNAP2/MAP2/NTSR1/PAFAH1B1/TRPV2/RASGRF1/TMEM108/RNF40
CC	Synapse part	17/141	918/18363	0.0007	0.049	ADORA1/SYT9/CTNND2/GAD2/KCND2/ARRB2/MAP2/MEF2C/NTSR1/COPS4/ADGRB3/SLC6A4/TMEM108/YWHAG/SYNGAP1/CLSTN3/RNF40
CC	Cytoplasmic region	11/141	473/18363	0.0011	0.059	AKT2/FGF1/COBL/SH3BP1/MAP2/MYH9/PAFAH1B1/CFAP46/TMEM108/ACTN4/ARHGEF7
CC	Distal axon	8/141	280/18363	0.0015	0.059	ADORA1/COBL/MAP2/NTSR1/PAFAH1B1/TRPV2/RASGRF1/RNF40
CC	Cell cortex	8/141	288/18363	0.0017	0.059	AKT2/FGF1/COBL/SH3BP1/MYH9/PAFAH1B1/ACTN4/ARHGEF7
CC	Actomyosin	4/141	71/18363	0.0022	0.059	MYH9/LURAP1/ACTN4/HDAC4
CC	Dendrite	12/141	602/18363	0.0024	0.059	ADORA1/CTNND2/COBL/CNTNAP2/KCNIP1/KCND2/ARRB2/MAP2/NTSR1/TMEM108/URI1/SYNGAP1
CC	Dendritic shaft	3/141	35/18363	0.0024	0.059	MAP2/NTSR1/SYNGAP1
CC	Dendritic tree	12/141	604/18363	0.0024	0.059	ADORA1/CTNND2/COBL/CNTNAP2/KCNIP1/KCND2/ARRB2/MAP2/NTSR1/TMEM108/URI1/SYNGAP1
CC	Postsynapse	12/141	604/18363	0.0024	0.059	ADORA1/CTNND2/KCND2/ARRB2/MAP2/MEF2C/NTSR1/ADGRB3/SLC6A4/TMEM108/SYNGAP1/CLSTN3
CC	Postsynaptic density	8/141	315/18363	0.0030	0.059	ADORA1/CTNND2/KCND2/ARRB2/MAP2/TMEM108/SYNGAP1/CLSTN3
CC	Cell leading edge	9/141	389/18363	0.0032	0.059	ABI2/ADORA1/AKT2/COBL/SH3BP1/CNTNAP2/MYH9/PAFAH1B1/ARHGEF7
CC	Cell body	11/141	545/18363	0.0033	0.059	ADORA1/CTNND2/COBL/CNTNAP2/KCND2/MAP2/NTSR1/PAFAH1B1/TRPV2/TCP1/ARHGEF7
CC	Asymmetric synapse	8/141	319/18363	0.0033	0.059	ADORA1/CTNND2/KCND2/ARRB2/MAP2/TMEM108/SYNGAP1/CLSTN3
CC	Somatodendritic compartment	14/141	818/18363	0.0042	0.069	ADORA1/CTNND2/COBL/CNTNAP2/KCNIP1/KCND2/ARRB2/MAP2/NTSR1/PAFAH1B1/TMEM108/URI1/SYNGAP1/ARHGEF7
CC	Dendrite terminus	2/141	13/18363	0.0043	0.069	COBL/MAP2
CC	Postsynaptic specialization	8/141	339/18363	0.0047	0.069	ADORA1/CTNND2/KCND2/ARRB2/MAP2/TMEM108/SYNGAP1/CLSTN3
CC	Voltage-gated potassium channel complex	4/141	89/18363	0.0049	0.069	CNTNAP2/KCNIP1/KCND2/KCNJ5
CC	Nucleotide-excision repair complex	2/141	14/18363	0.0050	0.069	CETN3/ERCC1
CC	Axolemma	2/141	15/18363	0.0058	0.076	ADORA1/CNTNAP2
CC	Axon	11/141	592/18363	0.0060	0.076	ADORA1/COBL/GAD2/CNTNAP2/MAP2/NTSR1/PAFAH1B1/TRPV2/RASGRF1/TMEM108/RNF40
CC	Potassium channel complex	4/141	98/18363	0.0069	0.083	CNTNAP2/KCNIP1/KCND2/KCNJ5
CC	Growth cone part	2/141	17/18363	0.0074	0.086	PAFAH1B1/TRPV2
CC	Growth cone	5/141	165/18363	0.0090	0.100	COBL/MAP2/PAFAH1B1/TRPV2/RASGRF1
CC	Site of polarized growth	5/141	167/18363	0.0094	0.101	COBL/MAP2/PAFAH1B1/TRPV2/RASGRF1

*BP* biological process, *CC* cellular component, *Bg ratio* background ratio

**Fig. 1:**
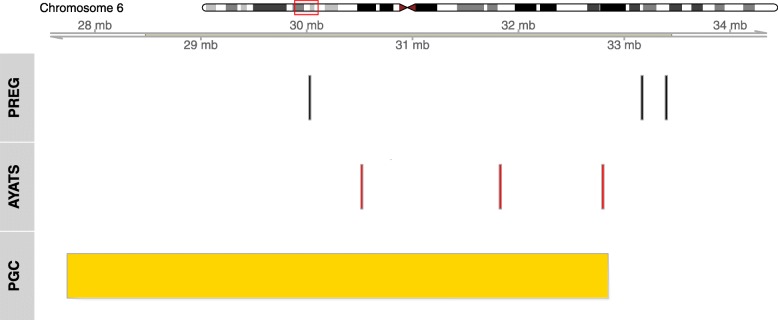
Significant differentially methylated regions on chromosome 6 overlap findings from a Psychiatric Genomics Consortium meta-analysis of depression. DNA methylation patterns associated with postpartum depressive symptoms (top row) and early-onset major depression (middle row) colocalize to the major histocompatibility complex (MHC) region on chromosome 6. The overlap of the DNA methylation patterns and the genomic region tagged in the genome-wide association meta-analysis of depression performed by the Psychiatric Genomics Consortium (PGC) is shown in the bottom row [58]

**Fig. 2:**
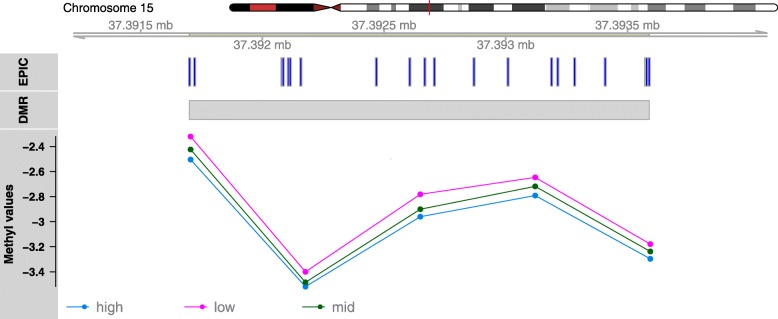
Increases in depressive symptom load negatively correlate with DNA methylation in a significant region identified on chromosome 15. The significantly differentially methylated region (row 2, “DMR”) was built using 5 of the 21 CpG probes available on the EPIC array (row 1, “EPIC”). The mean group methyl values are shown for low, mid, and high depressive symptom loads based on Edinburgh Postnatal Depression Scale (EPDS) total score (low = 0–4; mid = 5–12; high = 13 and greater). The threshold of 13 or greater for the high group was selected based on the validated cutoff score for English-speaking women in the postpartum time period shortly following birth [31]

Three DMRs overlapped PGC GWAS findings on chromosomes 5, 6, and 16 (*p* = 0.034; see Additional file 3 for more detail). The overlapping region on chromosome 6 occurred in the major histocompatibility complex (MHC) region and neighbored genomic areas previously associated with early-onset major depression (DNAm) [26] and depression as defined in the PGC genome-wide allelic meta-analysis (see Fig. 1) [57]. None of the sites or regions identified in the AYATS EWAS of early-onset major depression directly overlapped the DMRs associated with postpartum depressive symptom load in this analysis [26]. The DMR with the largest AUC was located on chromosome 15 and spanned five CpG sites (Fig. 2).

## Discussion

Evaluating the credibility and replicability of the significant findings from this analysis is paramount but complicated. Few directly comparable studies exist, and standardized practices for conducting and reporting results from epigenome-wide association studies (EWAS) have not been established. That said, domains to assess the credibility of EWAS results have been proposed, including the level of statistical significance, genomic location, biological relevance, functional relevance, validation of significant associations, and the potential for study design bias or confounding variables to influence the analysis [29]. An indirect effect of evaluating the results with these criteria is that it also highlights a study’s strengths and weaknesses.

This study identified 98 DMRs and 50 DMPs using DNAm measures from the EPIC beadchip (850 k), one of the most comprehensive microarray technologies available to assay DNAm. The combination strategy of site- and region-based analysis identified significant results that overlap a gene set enriched for biological pathways highly relevant to putative depression etiology (e.g., synaptic signaling and dendrite development). Moreover, the significant genome regions include a number of genes that have been previously associated with MD (e.g., *RNF145* [59]) or with estrogen and progesterone signaling (e.g., *FOXA1*, *ARRB2*, and *ITGB3BP*), which may be particularly relevant for MDP and perinatal depressive symptom risk liability [37]. None of the sites or regions from this directly overlapped regions significant in the AYATS EWAS of early-onset MD [26]; however, both this study and the AYATS EWAS identified significant DNAm regions in the major histocompatibility complex (MHC) region on chromosome 6, which was also identified in the latest PGC GWAS of depression [57]. The two DNAm studies also shared a large proportion of Gene Ontology (GO) terms in their respective gene set enrichment analyses (e.g., synaptic transmission and central nervous system development), suggesting that even if the exact sites differed by study, the biological pathways with associated genes did not.

Comparing these results within the DNAm-MDP literature is difficult because a majority of the studies used candidate gene approaches, which perform best as methods to refine results from genome-wide analyses. The only other genome-wide DNAm-MDP study that used maternal tissue measured prenatal DNAm with the HumanMethylation450 beadchip (450 k). No significant results were found, possibly due to a modest sample size (*n* = 38 antenatal maternal blood samples) [21] and using only single probe approach. Modest sample sizes are common in EWAS because of technology costs, but regional analyses can mitigate power issues from small sample sizes by reducing the multiple test burden. Ong and Holbrook estimated that using their regional approach, a two-group 450 k study with 38 people (*n* = 19 per group) would have 61% power to identify results with an effect size of 2 using a regional approach compared to 39% with a single probe analysis [30]. This power calculation does not map directly onto the analysis described here because Ong and Holbrook’s estimate is for a case-control study design. This study used a continuous measure of depression to avoid losing statistical power from dichotomizing a naturally quantitative trait. As a result, this study likely had at least 61% power but may not have had 80%. Another benefit of regional analyses is that the results are more likely to replicate [30] in part because significant regional results require multiple nearby probes each to have test statistics greater than a chosen threshold and to exhibit the same direction of effect. Together, the use of genome-wide DNAm, a permutation rank-based approach for assessing significance, and probes with test statistics either in the upper or lower 2.5 percentile to test for DMRs each positively influence the credibility of the results. Further, the genomic locations of the significant DMRs increase the credibility of the results as they overlap genes and genomic regions that either directly corroborate previous findings in the literature or that participate in biological pathways hypothesized to be important for depression risk or onset.

Another strength of this study concerns its design. Significant care was taken to minimize potential biases and confounders from biological, behavioral, and technical factors. The women in this study completed multiple comprehensive questionnaires about perinatal health and behaviors, accessed prenatal care relatively early in gestation, and were generally healthy (e.g., no diabetes) [28], had healthy singleton pregnancies, and completed a postpartum study visit within 7 months of delivery. These study design aspects allow variation from behavioral, biological, and technical factors to be measured and accounted for (e.g., cell type heterogeneity, tobacco use, slide and positional effects, and signal quality). Additionally, ancestrally-relevant principal components were incorporated to reduce the likelihood of detecting artifacts due to potential population stratification. Visualization of methyl values by self-reported race in significant DMRs suggested that DNAm values did not differ markedly between groups (see Additional file 4; Fig. 2).

Branching out to other depression phenotypes provides both more literature for comparison, but also more uncertainty. For example, it is not immediately clear if the DNAm patterns associated with postpartum depressive symptoms should resemble those associated with other depression phenotypes, including clinical major depression. Though both are depression phenotypes, depressive symptoms and clinical depression are not equivalent [60–63]. The issue of nonequivalence emerged when comparing the findings in this analysis to published results. For example, Numata et al. reported a significant relationship between the DNAm at cg14472315 and MD case status [25]. No significant relationship existed between that probe and self-reported postpartum depressive symptoms in this study; however, a nominally significant relationship between that probe and lifetime history of MD was present in this study (tested post hoc), reaffirming the difficulty of comparing studies with related but non-identical depressive phenotypes.

For all of its strengths, this study was not without limitations. First, the sample size was modest, which limits the statistical power to conduct robust single-site analysis. The sample also included relatively few women with severe levels of postpartum depressive symptoms, which may have unique DNAm signatures compared to clinical MDP. Moreover, this study could not determine if the DNAm patterns identified in this study were a consequence of previous episodes of MD (which is a risk factor for perinatal depression [46]) or if they are related to other genetic or environmental factors. Second, detailed information about medication history was not available. Third, the newness of the EPIC microarray means that the probe set has been less well-vetted for cross-hybridization compared to its predecessor [42, 64]. Four, regional analyses are inherently limited because not all probes have the potential to form regions [30]. This analysis attempted to address that weakness by also performing a single site analysis. Furthermore, the regional analysis algorithm used in this study selected an equal number of probes with positive and negative *t* values, corresponding to hypermethylation and hypomethylation in cases versus controls. This strategy assumes that equal representation of positive and negative *t* values will yield the most fair results; however, it is possible that this assumption limited the number of DMRs identified, especially if cases had much more hyper- or hypomethylation. Finally, this study was limited in its assessment of functional relevance. Multiple techniques were used to assess gene set enrichment, but none were specifically intended to correct for probe count per region, leading to differences in enrichment results by method. No gene expression, chromatin conformation, or transcription factor binding assays were run concurrently with postpartum DNAm analyses. No algorithms currently exist that can determine the precise change in DNAm necessary to translate into biologically meaningful differences in chromatin shape or gene regulation. As a result, fully understanding the etiology of MDP and depressive symptoms will likely involve integrating repeated measures from multiple biological layers (e.g., genetic sequence, epigenetic mechanisms, transcription, and protein) [65, 66], but no single study could measure every biological layer that might be informative about depression etiology, especially not longitudinally.

Another important consideration for interpreting DNAm results is the tissue source [29, 67]. It remains unclear how detrimental the use of peripheral blood DNAm is for identifying genomic regions associated with a psychiatric phenotype like depression. On one hand, specific brain regions hold intuitive appeal for MD-DNAm studies, and the cross-tissue similarity between brain and blood appears to be modest and tied to allelic variation [68]; however, given the well-established link between MD pathophysiology and aberrant immune system functioning [34–36, 69], peripheral blood may be the best and most feasible option for large or longitudinal studies of stress-related psychiatric traits. Many of the biomarkers associated with MD are transported in the blood (e.g., IL-1, IL-2, IL-6, TNFa, and haptoglobin), and some of these immune-related differences appear to persist after depressive episode remission. Not only does that observation fit with epidemiological studies that find individuals with a positive lifetime history of MD remain sensitive to stress and at higher risk for adverse auto-immune and cardiovascular outcomes [34], but also it suggests that DNAm patterns detectable in the blood may retain MD-associated differences even after depressive episode remission. Last but not least, peripheral blood may be useful for indexing changes in the relationship between the CNS and immune system. As a sentinel tissue, peripheral blood travels throughout the entire body and can deliver immune cells through the blood-brain barrier. Key players in the CNS like the neurotransmitter serotonin also serve roles in immune-related biological pathways (e.g., leukocyte activation and proliferation, cytokine secretion, chemotaxis, and apoptosis) [36, 70]. The ability of the CNS to modulate and respond to signals from the immune system underscores the intimate relationship between brain function and immune system regulation.

The final category Michels et al. (2013) listed as an important factor in establishing the credibility of DNAm associations with phenotypes is validation [29]. Validating results typically implies either replicating the finding in an independent study (human or animal) or confirming the presence of differentially methylated probes and regions using another technology (e.g., pyrosequencing). While no pyrosequencing was completed, the literature was searched extensively for associations between depressive phenotypes and biological signatures (e.g., allelic, epigenetic, and transcription). As previously mentioned, the genomic regions implicated in this study overlap results from the 2018 Psychiatric Genomics Consortium (PGC) genome-wide allelic association study (GWAS) of MD [57] and are proximal to significant regions and probes from an EWAS of early-onset MD. Network analysis of the 44 significant loci in the PGC meta-analysis implicated biological pathways associated with neural differentiation, synaptic regulation, risk for schizophrenia, immune response, ion-gated channels, and retinoid X receptors [57]. Similarly, this analysis identified significant DMRs overlapping genes coding for or related to retinoid X receptors (*RXRB*, *ITGB3BP*), genes integral to adult neurogenesis and synaptic development and positioning (e.g., *AKT2*, *SYNGAP1*, *FOXG1*, *CTNND2*, *MEF2C*, and *AIMP1*), risk for schizophrenia (e.g., *CLSTN3*, *FARSB*, *MYOM2*, *SOX2-OT*, *SLC39A7*, *SDCCAG8*, and *LRRC36*), immune response (*LRR1*, *FAM19A2*, *CMKLR1*, and *MMP2*), and ion-gated channels and binding (e.g., *SLC6A4*, *SLC6A12*, *SLC39A7*, *KCNJ5*, *CLSTN3*, and *PLS3*). While these similarities do not serve as direct replication, they do increase the credibility of the DNAm findings and underscore the potential for DNAm studies to complement GWA studies.

## Conclusions

Future work should take note of the apparent differences in depressive symptoms and clinical MD and seek to refine association studies by limiting phenotypic heterogeneity. For MDP, that means not only accounting for whether the depressive symptoms or episodes onset prenatally versus postnatally, but also disentangling which DNAm patterns are associated with perinatal depressive phenotypes and which reflect pre-pregnancy events of depressive psychopathology. It is possible that women who experience their first instance of MD in the peripartum have a unique DNAm profile compared to those who have recurrent MD and happen to onset during the peripartum. Furthermore, it is unknown whether an episode of MD relatively early in life evokes a persistent perturbation in DNAm patterning that then goes on to affect the risk for additional depressive symptoms and episodes as well as other adverse health outcomes frequently comorbid with depression (e.g., cardiovascular disease and diabetes mellitus). Clarifying the phenotypes associated with DNAm patterns will enable wet lab researchers to characterize the functional and biological relevance of implicated genomic sites and regions, which is an essential step not only for understanding the biological mechanisms associated with risk and resilience to depressive psychopathology but also for developing screening tests and identifying novel pharmacotherapeutic targets.

## Supplementary information

**Additional file 1.** DMP-only and DMR-only gene enrichment analysis. This file contains gene ontology enrichment results in tabular format for the combined set of differentially methylated regions and probes (DMR, DMP) results, enrichment analysis of DMRs only, and enrichment analysis of DMPs only. CSV files for these results are on the Open Science Framework (OSF; landing page https://osf.io/qsc6n).

**Additional file 2.** List of genes overlapping differentially methylated regions. List of the 92 genes overlapped by significant differentially methylated regions (DMRs).

**Additional file 3.** Enrichment testing of Psychiatric Genomic Consortium (PGC) supplemental methods. This file contains additional details about how the 95% confidence intervals were calculated for the PGC enrichment analysis.

**Additional file 4:** Supplement to Figure 2. This file contains two additional versions of the differentially methylated region (DMR) highlighted in Fig. 2. The top figure shows the ComBat-adjusted methyl values for each participant colored by self-identified Census-based race category. The points have been jittered left/right to ease visualization by reducing over-plotting. No vertical adjustment was made. The bottom figure shows the mean methyl values for each probe contained in the DMR by self-identified Census-based race category.

**Additional file 5.** Additional Figures. This file contains four figures: quantile-quantile plots for the DMP and DMR analyses and histograms of the distribution of when PREG postpartum study visits occurred (i.e., time since birth) and the distribution of EPDS total scores by self-reported lifetime history of MD (assessed using an extended self-report version of the CIDI-SF).

## Data Availability

The PREG study research team is committed to participating in open science practices as much as possible. For this specific dataset, the IRB agreements and consent forms restrict the availability of the data due to privacy concerns. These precautions were taken as part of an effort to respect the autonomy and privacy and to encourage individuals from minority populations to feel comfortable participating in racial health disparities research. Anyone interested in access to data from participants who consented to permit data sharing is encouraged to contact Dr. Timothy P. York (corresponding author). Summary-level data and methods can be found on the Open Science Framework (OSF; landing page https://osf.io/qsc6n). Summary statistics for individual probes and regions can be found at https://osf.io/q2s8d. Detailed information about specimen quality control assessments and covariate selection also are available on the project page in the Supplementary Information component (https://osf.io/8p9q6).

## Abbreviations

AYATS: Adolescent and Young Adult Twin Study
CpG: Cytosine-phosphate-guanine
DMP: Differentially methylated probe
DMR: Differentially methylated region
DNAm: DNA methylation
EPDS: Edinburgh Postnatal Depression Scale
EWAS: Epigenome-wide association study
FDR: False discovery rate
GO: Gene Ontology
GWAS: Genome-wide association study
MD: Major depression
MDP: Major depression with onset in the peripartum
MHC: Major histocompatibility complex
PCA/PC: Principal components analysis/principal component
PGC: Psychiatric Genomics Consortium
PREG: Pregnancy, Race, Environment, Genes study
SNP: Single-nucleotide polymorphism
