# Supplementary material for: DNA methylation associated with postpartum depressive symptoms overlaps findings from a genome-wide association meta-analysis of depression
Source: Clin Epigenetics. 2019 Nov 28;11:169. doi: 10.1186/s13148-019-0769-z (PMC6883636; doi:10.1186/s13148-019-0769-z)
Supplement: Supplementary file 1 — Additional file 1. DMP-only and DMR-only gene enrichment analysis. This file contains gene ontology enrichment results in tabular format for the combined set of differentially methylated regions and probes (DMR, DMP) results, enrichment analysis of DMRs only, and enrichment analysis of DMPs only. CSV files for these results are on the Open Science Framework (OSF; landing page https://osf.io/qsc6n). [file 13148_2019_769_MOESM1_ESM.pdf]

Gene Ontology Supplemental Tables

Summary

This file contains the full results for the combined gene set enrichment analysis and the DMR and DMP only enrichment analyses performed with clusterProfiler. DMP enrichment tests also were performed with methylGSA.

clusterProfiler

DMR & DMP

Ontology	Description	GeneRatio	BgRatio	pvalue	qvalue	geneID
BP	cognition	11/136	284/17397	0.0000	0.038	TTC8/ADORA1/CNTNAP2/MEF2C/MEIS2/NTSR1/PAFAH1B1/ ADGRB3/RASGRF1/SLC6A4/SYNGAP1
BP	learning or memory	9/136	246/17397	0.0001	0.114	CNTNAP2/MEF2C/MEIS2/NTSR1/PAFAH1B1/ADGRB3/RASGRF1/ SLC6A4/SYNGAP1
BP	detection of temperature stimulus i	3/136	15/17397	0.0002	0.114	ADORA1/ARRB2/NTSR1
BP	detection of temperature stimulus i	3/136	15/17397	0.0002	0.114	ADORA1/ARRB2/NTSR1

BP	dendrite development	8/136	216/17397	0.0003	0.114	CTNND2/COBL/MAP2/MEF2C/PAFAH1B1/ADGRB3/KLF7/SYNGAP1
BP	detection of temperature stimulus	3/136	19/17397	0.0004	0.114	ADORA1/ARRB2/NTSR1
BP	modulation of chemical synaptic tra	11/136	417/17397	0.0004	0.114	ADORA1/SYT9/ARRB2/MEF2C/NTSR1/RASGRF1/SLC6A4/TMEM108/YWHAG/SYNGAP1/CLSTN3
BP	regulation of trans-synaptic signal	11/136	418/17397	0.0004	0.114	ADORA1/SYT9/ARRB2/MEF2C/NTSR1/RASGRF1/SLC6A4/TMEM108/YWHAG/SYNGAP1/CLSTN3
BP	platelet formation	3/136	20/17397	0.0005	0.114	ZFPM1/MEF2C/MYH9
BP	establishment of cell polarity	6/136	128/17397	0.0005	0.114	SDCCAG8/SH3BP1/MAP2/MYH9/PAFAH1B1/FRMD4A
BP	actin filament-based process	15/136	723/17397	0.0005	0.114	ABI2/ADORA1/DIAPH2/COBL/SH3BP1/KCNJ5/MEF2C/MYH9/NEB/PAFAH1B1/PLS3/LURAP1/ACTN4/MYOM2/TBCK
BP	platelet morphogenesis	3/136	21/17397	0.0006	0.114	ZFPM1/MEF2C/MYH9
BP	sensory perception of temperature s	3/136	21/17397	0.0006	0.114	ADORA1/ARRB2/NTSR1
BP	behavior	13/136	579/17397	0.0006	0.116	ADORA1/CNTNAP2/KCND2/ARRB2/MEF2C/MEIS2/NTSR1/PAFAH1B1/PEX13/ADGRB3/RASGRF1/SLC6A4/SYNGAP1
BP	response to hypoxia	9/136	308/17397	0.0007	0.125	ADORA1/HILPDA/KCND2/LMNA/MMP2/HIF3A/SLC6A4/TGFBR2/ACTN4
BP	regulation of postsynaptic membrane	6/136	139/17397	0.0008	0.128	ADORA1/KCND2/ARRB2/MEF2C/NTSR1/TMEM108
BP	response to decreased oxygen levels	9/136	319/17397	0.0009	0.128	ADORA1/HILPDA/KCND2/LMNA/MMP2/HIF3A/SLC6A4/TGFBR2/ACTN4

BP	chemical synaptic transmission	14/136	685/17397	0.0010	0.128	ADORA1/SYT9/GAD2/KCND2/ARRB2/MEF2C/NTSR1/PAFAH1B1/RASGRF1/SLC6A4/TMEM108/YWHAG/SYNGAP1/CLSTN3
BP	anterograde trans-synaptic signalin	14/136	685/17397	0.0010	0.128	ADORA1/SYT9/GAD2/KCND2/ARRB2/MEF2C/NTSR1/PAFAH1B1/RASGRF1/SLC6A4/TMEM108/YWHAG/SYNGAP1/CLSTN3
BP	establishment or maintenance of cel	7/136	199/17397	0.0010	0.128	SDCCAG8/SH3BP1/LMNA/MAP2/MYH9/PAFAH1B1/FRMD4A
BP	trans-synaptic signaling	14/136	693/17397	0.0011	0.134	ADORA1/SYT9/GAD2/KCND2/ARRB2/MEF2C/NTSR1/PAFAH1B1/RASGRF1/SLC6A4/TMEM108/YWHAG/SYNGAP1/CLSTN3
BP	synaptic signaling	14/136	698/17397	0.0011	0.137	ADORA1/SYT9/GAD2/KCND2/ARRB2/MEF2C/NTSR1/PAFAH1B1/RASGRF1/SLC6A4/TMEM108/YWHAG/SYNGAP1/CLSTN3
BP	striated muscle cell differentiatio	8/136	269/17397	0.0013	0.140	LMNA/ARRB2/MEF2C/MYH9/NEB/ADGRB3/MYOM2/HDAC4
BP	actin cytoskeleton organization	13/136	633/17397	0.0014	0.140	ABI2/DIAPH2/COBL/SH3BP1/MEF2C/MYH9/NEB/PAFAH1B1/PLS3/LURAP1/ACTN4/MYOM2/TBCK
BP	muscle hypertrophy in response to s	3/136	29/17397	0.0015	0.140	LMNA/MEF2C/HDAC4
BP	cardiac muscle adaptation	3/136	29/17397	0.0015	0.140	LMNA/MEF2C/HDAC4
BP	cardiac muscle hypertrophy in respo	3/136	29/17397	0.0015	0.140	LMNA/MEF2C/HDAC4
BP	response to oxygen levels	9/136	342/17397	0.0015	0.140	ADORA1/HILPDA/KCND2/LMNA/MMP2/HIF3A/SLC6A4/TGFBR2/ACTN4
BP	cardiac muscle tissue development	7/136	216/17397	0.0016	0.142	ZFPM1/LMNA/ARRB2/MEF2C/NEB/TGFBR2/MYOM2

BP	supramolecular fiber organization	13/136	645/17397	0.0016	0.144	ABI2/CETN3/DIAPH2/COBL/SH3BP1/FIGNL2/MAP2/MEF2C/NEB/PAFAH1B1/PLS3/ACTN4/MYOM2
CC	DNA repair complex	4/141	42/18363	0.0003	0.044	CETN3/ERCC1/PAXX/WRN
CC	neuron to neuron synapse	10/141	340/18363	0.0003	0.044	ADORA1/SYT9/CTNND2/KCND2/ARRB2/MAP2/NTSR1/TMEM108/SYNGAP1/CLSTN3
CC	axon part	10/141	373/18363	0.0006	0.049	ADORA1/COBL/CNTNAP2/MAP2/NTSR1/PAFAH1B1/TRPV2/RASGRF1/TMEM108/RNF40
CC	synapse part	17/141	918/18363	0.0007	0.049	ADORA1/SYT9/CTNND2/GAD2/KCND2/ARRB2/MAP2/MEF2C/NTSR1/COPS4/ADGRB3/SLC6A4/TMEM108/YWHAG/SYNGAP1/CLSTN3/RNF40
CC	cytoplasmic region	11/141	473/18363	0.0011	0.059	AKT2/FGF1/COBL/SH3BP1/MAP2/MYH9/PAFAH1B1/CFAP46/TMEM108/ACTN4/ARHGEF7
CC	distal axon	8/141	280/18363	0.0015	0.059	ADORA1/COBL/MAP2/NTSR1/PAFAH1B1/TRPV2/RASGRF1/RNF40
CC	cell cortex	8/141	288/18363	0.0017	0.059	AKT2/FGF1/COBL/SH3BP1/MYH9/PAFAH1B1/ACTN4/ARHGEF7
CC	actomyosin	4/141	71/18363	0.0022	0.059	MYH9/LURAP1/ACTN4/HDAC4
CC	dendrite	12/141	602/18363	0.0024	0.059	ADORA1/CTNND2/COBL/CNTNAP2/KCNIP1/KCND2/ARRB2/MAP2/NTSR1/TMEM108/URI1/SYNGAP1
CC	dendritic shaft	3/141	35/18363	0.0024	0.059	MAP2/NTSR1/SYNGAP1
CC	dendritic tree	12/141	604/18363	0.0024	0.059	ADORA1/CTNND2/COBL/CNTNAP2/KCNIP1/KCND2/ARRB2/MAP2/NTSR1/TMEM108/URI1/SYNGAP1
CC	postsynapse	12/141	604/18363	0.0024	0.059	ADORA1/CTNND2/KCND2/ARRB2/MAP2/MEF2C/NTSR1/ADGRB3/SLC6A4/TMEM108/SYNGAP1/CLSTN3
CC	postsynaptic density	8/141	315/18363	0.0030	0.059	ADORA1/CTNND2/KCND2/ARRB2/MAP2/TMEM108/SYNGAP1/CLSTN3
CC	cell leading edge	9/141	389/18363	0.0032	0.059	ABI2/ADORA1/AKT2/COBL/SH3BP1/CNTNAP2/MYH9/PAFAH1B1/ARHGEF7
CC	cell body	11/141	545/18363	0.0033	0.059	ADORA1/CTNND2/COBL/CNTNAP2/KCND2/MAP2/NTSR1/PAFAH1B1/TRPV2/TCP1/ARHGEF7

CC	asymmetric synapse	8/141	319/18363	0.0033	0.059	ADORA1/CTNND2/KCND2/ARRB2/MAP2/TMEM108/SYNGAP1/CLSTN3
CC	somatodendritic compartment	14/141	818/18363	0.0042	0.069	ADORA1/CTNND2/COBL/CNTNAP2/KCNIP1/KCND2/ARRB2/MAP2/NTSR1/PAFAH1B1/TMEM108/URI1/SYNGAP1/ARHGEF7
CC	dendrite terminus	2/141	13/18363	0.0043	0.069	COBL/MAP2
CC	postsynaptic specialization	8/141	339/18363	0.0047	0.069	ADORA1/CTNND2/KCND2/ARRB2/MAP2/TMEM108/SYNGAP1/CLSTN3
CC	voltage-gated potassium channel com	4/141	89/18363	0.0049	0.069	CNTNAP2/KCNIP1/KCND2/KCNJ5
CC	nucleotide-excision repair complex	2/141	14/18363	0.0050	0.069	CETN3/ERCC1
CC	axolemma	2/141	15/18363	0.0058	0.076	ADORA1/CNTNAP2
CC	axon	11/141	592/18363	0.0060	0.076	ADORA1/COBL/GAD2/CNTNAP2/MAP2/NTSR1/PAFAH1B1/TRPV2/RASGRF1/TMEM108/RNF40
CC	potassium channel complex	4/141	98/18363	0.0069	0.083	CNTNAP2/KCNIP1/KCND2/KCNJ5
CC	growth cone part	2/141	17/18363	0.0074	0.086	PAFAH1B1/TRPV2
CC	growth cone	5/141	165/18363	0.0090	0.100	COBL/MAP2/PAFAH1B1/TRPV2/RASGRF1
CC	site of polarized growth	5/141	167/18363	0.0094	0.101	COBL/MAP2/PAFAH1B1/TRPV2/RASGRF1

DMR only

Ontology	Description	GeneRatio	BgRatio	pvalue	qvalue	geneID
BP	platelet formation	3/97	20/17137	0.0002	0.232	ZFPM1/MEF2C/MYH9
BP	platelet morphogenesis	3/97	21/17137	0.0002	0.232	ZFPM1/MEF2C/MYH9
BP	cardiac muscle tissue development	6/97	214/17137	0.0014	0.410	ZFPM1/LMNA/ARRB2/MEF2C/TGFBR2/MYOM2
BP	regulation of macrophage apoptoti	2/97	10/17137	0.0014	0.410	MEF2C/TCP1
BP	macrophage apoptotic process	2/97	12/17137	0.0020	0.410	MEF2C/TCP1
BP	heart valve morphogenesis	3/97	47/17137	0.0024	0.410	ZFPM1/MEF2C/TGFBR2
BP	actin filament-based process	11/97	721/17137	0.0025	0.410	ABI2/ADORA1/DIAPH2/SH3BP1/KCNJ5/MEF2C/MYH9/PLS3/LURAP1/MYOM2/TBCK
BP	excitatory postsynaptic potential	4/97	103/17137	0.0028	0.410	ADORA1/ARRB2/MEF2C/TMEM108
BP	lung morphogenesis	3/97	50/17137	0.0028	0.410	FOXA1/HHIP/TGFBR2
BP	negative regulation of interleuki	2/97	15/17137	0.0032	0.410	CMKLR1/ARRB2
BP	detection of temperature stimulus	2/97	15/17137	0.0032	0.410	ADORA1/ARRB2
BP	detection of temperature stimulus	2/97	15/17137	0.0032	0.410	ADORA1/ARRB2
BP	heart valve development	3/97	53/17137	0.0034	0.410	ZFPM1/MEF2C/TGFBR2
BP	regulation of synaptic transmissi	2/97	16/17137	0.0036	0.410	ARRB2/SLC6A4
BP	chemical synaptic transmission, p	4/97	111/17137	0.0036	0.410	ADORA1/ARRB2/MEF2C/TMEM108

BP	positive regulation of intracellu	2/97	17/17137	0.0041	0.410	FOXA1/LMO3
BP	ventricular cardiac muscle cell d	2/97	17/17137	0.0041	0.410	LMNA/MEF2C
BP	striated muscle cell differentiat	6/97	268/17137	0.0042	0.410	LMNA/ARRB2/MEF2C/MYH9/ADGRB3/MYOM2
BP	striated muscle tissue developmen	7/97	367/17137	0.0048	0.410	ZFPM1/LMNA/ARRB2/MEF2C/HIVEP3/TGFBR2/MYOM2
BP	animal organ formation	3/97	61/17137	0.0050	0.410	FGF1/MEF2C/TGFBR2
BP	detection of temperature stimulus	2/97	19/17137	0.0051	0.410	ADORA1/ARRB2
BP	negative regulation of release of	2/97	19/17137	0.0051	0.410	LMNA/ARRB2
BP	cardiac muscle cell differentiati	4/97	123/17137	0.0052	0.410	LMNA/ARRB2/MEF2C/MYOM2
BP	establishment or maintenance of c	5/97	199/17137	0.0055	0.410	SDCCAG8/SH3BP1/LMNA/MYH9/FRMD4A
BP	cognition	6/97	284/17137	0.0055	0.410	TTC8/ADORA1/MEF2C/MEIS2/ADGRB3/SLC6A4
BP	negative regulation of organic ac	2/97	20/17137	0.0056	0.410	ADORA1/AKT2
BP	muscle tissue development	7/97	382/17137	0.0060	0.410	ZFPM1/LMNA/ARRB2/MEF2C/HIVEP3/TGFBR2/MYOM2
BP	establishment of cell polarity	4/97	128/17137	0.0060	0.410	SDCCAG8/SH3BP1/MYH9/FRMD4A
BP	embryonic hemopoiesis	2/97	21/17137	0.0062	0.410	ZFPM1/TGFBR2
BP	sensory perception of temperature	2/97	21/17137	0.0062	0.410	ADORA1/ARRB2
BP	atrioventricular valve morphogene	2/97	22/17137	0.0068	0.410	ZFPM1/TGFBR2

BP	inflammatory cell apoptotic proce	2/97	22/17137	0.0068	0.410	MEF2C/TCP1
BP	negative regulation of endothelia	3/97	69/17137	0.0070	0.410	MIR129-2/MEF2C/AIMP1
BP	positive regulation of lipid cata	2/97	23/17137	0.0074	0.410	ADORA1/AKT2
BP	tissue morphogenesis	9/97	608/17137	0.0075	0.410	ZFPM1/FGF1/SH3BP1/FOXA1/ARRB2/MEF2C/HHIP/TGFBR2/MYOM2
BP	regulation of postsynaptic membra	4/97	139/17137	0.0080	0.410	ADORA1/ARRB2/MEF2C/TMEM108
BP	atrioventricular valve developmen	2/97	24/17137	0.0081	0.410	ZFPM1/TGFBR2
BP	negative regulation of vascular e	2/97	24/17137	0.0081	0.410	MIR129-2/MEF2C
BP	outflow tract morphogenesis	3/97	76/17137	0.0092	0.410	ZFPM1/MEF2C/TGFBR2
BP	negative regulation of anion tran	2/97	26/17137	0.0094	0.410	ADORA1/AKT2
BP	actin cytoskeleton organization	9/97	631/17137	0.0095	0.410	ABI2/DIAPH2/SH3BP1/MEF2C/MYH9/PLS3/LURAP1/MYOM2/TBCK
MF	ATP-dependent helicase activity	3/93	73/16598	0.0080	0.402	CHD3/WRN/DHX38
MF	purine NTP-dependent helicase act	3/93	73/16598	0.0080	0.402	CHD3/WRN/DHX38
MF	neurotransmitter:sodium symporter	2/93	27/16598	0.0100	0.402	SLC6A4/SLC6A12
CC	DNA repair complex	3/100	41/18080	0.0015	0.443	CETN3/PAXX/WRN
CC	myosin filament	2/100	22/18080	0.0065	0.475	MYH9/MYOM2

DMP only

Ontology	Description	GeneRatio	BgRatio	pvalue	qvalue	geneID
BP	central nervous system neuron dev	3/38	69/16678	0.0005	0.238	DCLK2/MAP2/PAFAH1B1
BP	transmission of nerve impulse	3/38	71/16678	0.0006	0.238	CNTNAP2/KCND2/PAFAH1B1
BP	central nervous system neuron dif	4/38	170/16678	0.0006	0.238	DCLK2/MAP2/PAFAH1B1/WNT9B
BP	multicellular organismal signalin	4/38	199/16678	0.0011	0.245	CNTNAP2/KCNIP1/KCND2/PAFAH1B1
BP	limbic system development	3/38	101/16678	0.0016	0.245	DCLK2/CNTNAP2/PAFAH1B1
BP	learning or memory	4/38	233/16678	0.0019	0.245	CNTNAP2/NTSR1/PAFAH1B1/RASGRF1
BP	chemical synaptic transmission	6/38	603/16678	0.0022	0.245	SYT9/KCND2/NTSR1/PAFAH1B1/RASGRF1/YWHAG
BP	anterograde trans-synaptic signal	6/38	603/16678	0.0022	0.245	SYT9/KCND2/NTSR1/PAFAH1B1/RASGRF1/YWHAG
BP	synaptic signaling	6/38	605/16678	0.0023	0.245	SYT9/KCND2/NTSR1/PAFAH1B1/RASGRF1/YWHAG
BP	trans-synaptic signaling	6/38	605/16678	0.0023	0.245	SYT9/KCND2/NTSR1/PAFAH1B1/RASGRF1/YWHAG
BP	establishment of cell polarity	3/38	120/16678	0.0026	0.245	MAP2/PAFAH1B1/FRMD4A
BP	phosphatidylcholine biosynthetic	2/38	36/16678	0.0030	0.245	PEMT/CHKA
BP	regulation of ion transmembrane t	5/38	440/16678	0.0031	0.245	KCNIP1/KCND2/NTSR1/RASGRF1/ACTN4
BP	cognition	4/38	270/16678	0.0032	0.245	CNTNAP2/NTSR1/PAFAH1B1/RASGRF1
BP	regulation of	5/38	455/16678	0.0035	0.245	KCNIP1/KCND2/NTSR1/RASGRF1/ACTN4

	transmembrane trans					
BP	adult behavior	3/38	140/16678	0.0039	0.245	CNTNAP2/NTSR1/PAFAH1B1
BP	regulation of cation transmembran	4/38	287/16678	0.0040	0.245	KCNIP1/NTSR1/RASGRF1/ACTN4
BP	vesicle cytoskeletal trafficking	2/38	43/16678	0.0043	0.245	PAFAH1B1/ACTN4
BP	pallium development	3/38	164/16678	0.0061	0.245	DCLK2/CNTNAP2/PAFAH1B1
BP	establishment or maintenance of c	3/38	182/16678	0.0081	0.245	MAP2/PAFAH1B1/FRMD4A
BP	behavior	5/38	574/16678	0.0093	0.245	CNTNAP2/KCND2/NTSR1/PAFAH1B1/RASGRF1
BP	locomotory behavior	3/38	192/16678	0.0094	0.245	KCND2/NTSR1/PAFAH1B1
BP	ammonium ion metabolic process	3/38	194/16678	0.0097	0.245	PEMT/CHKA/PAFAH1B1
BP	positive regulation of dendrite d	2/38	66/16678	0.0099	0.245	COBL/PAFAH1B1
CC	dendrite	7/40	492/17666	0.0001	0.009	COBL/CNTNAP2/KCNIP1/KCND2/MAP2/NTSR1/URI1
CC	somatodendritic compartment	8/40	690/17666	0.0001	0.009	COBL/CNTNAP2/KCNIP1/KCND2/MAP2/NTSR1/PAFAH1B1/URI1
CC	neuronal cell body	6/40	435/17666	0.0004	0.014	COBL/CNTNAP2/KCND2/MAP2/NTSR1/PAFAH1B1
CC	axon part	4/40	180/17666	0.0007	0.014	COBL/CNTNAP2/NTSR1/PAFAH1B1
CC	supramolecular fiber	8/40	894/17666	0.0008	0.014	DCLK2/COBL/KRTAP19-4/MAP2/NEB/PAFAH1B1/ACTN4/HDAC4
CC	supramolecular polymer	8/40	901/17666	0.0008	0.014	DCLK2/COBL/KRTAP19-4/MAP2/NEB/PAFAH1B1/ACTN4/HDAC4
CC	supramolecular complex	8/40	902/17666	0.0008	0.014	DCLK2/COBL/KRTAP19-4/MAP2/NEB/PAFAH1B1/ACTN4/HDAC4

CC	cell body	6/40	499/17666	0.0008	0.014	COBL/CNTNAP2/KCND2/MAP2/NTSR1/PAFAH1B1
CC	voltage-gated potassium channel c	3/40	94/17666	0.0012	0.019	CNTNAP2/KCNIP1/KCND2
CC	potassium channel complex	3/40	98/17666	0.0014	0.019	CNTNAP2/KCNIP1/KCND2
CC	perikaryon	3/40	114/17666	0.0022	0.027	CNTNAP2/KCND2/NTSR1
CC	Z disc	3/40	119/17666	0.0025	0.028	NEB/ACTN4/HDAC4
CC	dendritic shaft	2/40	36/17666	0.0030	0.031	MAP2/NTSR1
CC	I band	3/40	132/17666	0.0033	0.032	NEB/ACTN4/HDAC4
CC	cluster of actin-based cell proje	3/40	140/17666	0.0039	0.035	PEMT/PAFAH1B1/ACTN4
CC	growth cone	3/40	151/17666	0.0048	0.040	COBL/PAFAH1B1/RASGRF1
CC	site of polarized growth	3/40	156/17666	0.0053	0.042	COBL/PAFAH1B1/RASGRF1
CC	actomyosin	2/40	64/17666	0.0092	0.066	ACTN4/HDAC4
CC	sarcomere	3/40	192/17666	0.0093	0.066	NEB/ACTN4/HDAC4

methylGSA

DMP only Gene Ontology Enrichment

Gene set min/max limits used were 25/500 (`methylglm()`).

ID	Description	Size	pvalue	padj
GO:0032266	phosphatidylinositol-3-phosphate bi	45	0.0000045	0.0240348
GO:0050855	regulation of B cell receptor signa	26	0.0000790	0.2088900
GO:0008527	taste receptor activity	58	0.0003370	0.5932192
GO:0046889	positive regulation of lipid biosyn	84	0.0006970	0.9208960
GO:0015813	L-glutamate transmembrane transport	29	0.0009180	0.9705038
GO:0042165	neurotransmitter binding	71	0.0012677	1.0000000
GO:0080025	phosphatidylinositol-3,5-bisphospha	31	0.0013320	1.0000000
GO:0002832	negative regulation of response to	45	0.0015415	1.0000000
GO:0006699	bile acid biosynthetic process	53	0.0018560	1.0000000
GO:0008307	structural constituent of muscle	48	0.0019660	1.0000000
GO:0033038	bitter taste receptor activity	40	0.0021503	1.0000000
GO:0016460	myosin II complex	25	0.0027860	1.0000000
GO:0051213	dioxygenase activity	171	0.0028474	1.0000000
GO:0016706	oxidoreductase activity, acting on	91	0.0036439	1.0000000
GO:0045505	dynein intermediate chain binding	31	0.0047951	1.0000000
GO:0007585	respiratory gaseous exchange	66	0.0056135	1.0000000
GO:0005313	L-glutamate transmembrane transport	25	0.0058690	1.0000000
GO:0015172	acidic amino acid transmembrane tra	27	0.0058690	1.0000000
GO:0015740	C4-dicarboxylate transport	34	0.0061170	1.0000000
GO:1905898	positive regulation of response to	44	0.0062962	1.0000000
GO:0015807	L-amino acid transport	91	0.0066003	1.0000000

GO:0008277	regulation of G protein-coupled rec	162	0.0076417	1.0000000
GO:0045833	negative regulation of lipid metabo	104	0.0078834	1.0000000
GO:0051055	negative regulation of lipid biosyn	63	0.0088589	1.0000000
GO:0045834	positive regulation of lipid metabo	156	0.0090897	1.0000000
GO:0043303	mast cell degranulation	47	0.0091630	1.0000000
GO:0008106	alcohol dehydrogenase (NADP+) activ	30	0.0092378	1.0000000
GO:0045576	mast cell activation	62	0.0094712	1.0000000
GO:0002448	mast cell mediated immunity	49	0.0096261	1.0000000
GO:0046164	alcohol catabolic process	62	0.0096887	1.0000000
GO:0043304	regulation of mast cell degranulati	30	0.0099759	1.0000000
GO:0016056	rhodopsin mediated signaling pathwa	31	0.0105390	1.0000000
GO:1904315	transmitter-gated ion channel activ	62	0.0109396	1.0000000
GO:0006984	ER-nucleus signaling pathway	59	0.0114549	1.0000000
GO:0008206	bile acid metabolic process	68	0.0114689	1.0000000
GO:0002279	mast cell activation involved in im	49	0.0117326	1.0000000
GO:0071216	cellular response to biotic stimulu	264	0.0122937	1.0000000
GO:0007603	phototransduction, visible light	37	0.0125515	1.0000000
GO:0033006	regulation of mast cell activation	32	0.0140795	1.0000000
GO:0007602	phototransduction	68	0.0144581	1.0000000
GO:0016558	protein import into peroxisome matr	28	0.0145371	1.0000000
GO:0031935	regulation of chromatin silencing	31	0.0145857	1.0000000
GO:0035510	DNA dealkylation	52	0.0146308	1.0000000
GO:0022824	transmitter-gated ion channel activ	127	0.0149068	1.0000000
GO:0022835	transmitter-gated channel activity	127	0.0149068	1.0000000
GO:0050853	B cell receptor signaling pathway	130	0.0150774	1.0000000
GO:1900120	regulation of receptor binding	27	0.0151624	1.0000000

GO:1900181	negative regulation of protein loca	30	0.0156276	1.0000000
GO:0080111	DNA demethylation	42	0.0177546	1.0000000
GO:0015919	peroxisomal membrane transport	33	0.0178711	1.0000000
GO:0098960	postsynaptic neurotransmitter recep	69	0.0180256	1.0000000
GO:0042634	regulation of hair cycle	28	0.0187764	1.0000000
GO:0050687	negative regulation of defense resp	25	0.0188528	1.0000000
GO:0006865	amino acid transport	228	0.0193140	1.0000000
GO:0001825	blastocyst formation	30	0.0193539	1.0000000
GO:0010828	positive regulation of glucose tran	42	0.0195698	1.0000000
GO:0002228	natural killer cell mediated immuni	77	0.0202440	1.0000000
GO:0042056	chemoattractant activity	41	0.0208818	1.0000000
GO:0032418	lysosome localization	72	0.0212311	1.0000000
GO:0099529	neurotransmitter receptor activity	65	0.0215561	1.0000000
GO:0004659	prenyltransferase activity	32	0.0226063	1.0000000
GO:0043484	regulation of RNA splicing	185	0.0230393	1.0000000
GO:0016896	exoribonuclease activity, producing	60	0.0234295	1.0000000
GO:0005231	excitatory extracellular ligand-gat	60	0.0239671	1.0000000
GO:0051568	histone H3-K4 methylation	67	0.0240898	1.0000000
GO:0060090	molecular adaptor activity	213	0.0246617	1.0000000
GO:0003333	amino acid transmembrane transport	120	0.0250417	1.0000000
GO:0015179	L-amino acid transmembrane transpor	85	0.0260452	1.0000000
GO:0055013	cardiac muscle cell development	96	0.0261266	1.0000000
GO:0001578	microtubule bundle formation	117	0.0276591	1.0000000
GO:0070988	demethylation	103	0.0280041	1.0000000
GO:0030674	protein binding, bridging	181	0.0280907	1.0000000
GO:0044295	axonal growth cone	27	0.0284007	1.0000000

GO:0005230	extracellular ligand-gated ion chan	193	0.0287956	1.0000000
GO:0071482	cellular response to light stimulus	133	0.0306123	1.0000000
GO:0042269	regulation of natural killer cell m	52	0.0322845	1.0000000
GO:0051972	regulation of telomerase activity	59	0.0323730	1.0000000
GO:0098562	cytoplasmic side of membrane	193	0.0325306	1.0000000
GO:0005326	neurotransmitter transporter activi	84	0.0332505	1.0000000
GO:0055003	cardiac myofibril assembly	30	0.0337483	1.0000000
GO:0002675	positive regulation of acute inflam	28	0.0339626	1.0000000
GO:0043901	negative regulation of multi-organi	196	0.0340928	1.0000000
GO:1901981	phosphatidylinositol phosphate bind	184	0.0341943	1.0000000
GO:0046329	negative regulation of JNK cascade	41	0.0343055	1.0000000
GO:0002715	regulation of natural killer cell m	55	0.0346331	1.0000000
GO:0032958	inositol phosphate biosynthetic pro	29	0.0347975	1.0000000
GO:0060251	regulation of glial cell proliferat	34	0.0359224	1.0000000
GO:2000050	regulation of non-canonical Wnt sig	29	0.0359604	1.0000000
GO:0006835	dicarboxylic acid transport	115	0.0361361	1.0000000
GO:0046326	positive regulation of glucose impo	35	0.0362885	1.0000000
GO:0005776	autophagosome	125	0.0376970	1.0000000
GO:0001221	transcription cofactor binding	44	0.0386562	1.0000000
GO:0032206	positive regulation of telomere mai	64	0.0387354	1.0000000
GO:0071013	catalytic step 2 spliceosome	120	0.0388125	1.0000000
GO:0045746	negative regulation of Notch signal	44	0.0389699	1.0000000
GO:0032722	positive regulation of chemokine pr	58	0.0393304	1.0000000
GO:0015171	amino acid transmembrane transporte	154	0.0393704	1.0000000
GO:0036002	pre-mRNA binding	67	0.0423030	1.0000000
GO:0048024	regulation of mRNA splicing, via sp	134	0.0431612	1.0000000

GO:0071214	cellular response to abiotic stimul	361	0.0435137	1.0000000
GO:0104004	cellular response to environmental	361	0.0435137	1.0000000
GO:0045598	regulation of fat cell differentiat	132	0.0437368	1.0000000
GO:0044766	multi-organism transport	34	0.0437863	1.0000000
GO:1902579	multi-organism localization	34	0.0437863	1.0000000
GO:0005416	amino acid:cation symporter activit	33	0.0439685	1.0000000
GO:0034976	response to endoplasmic reticulum s	410	0.0448217	1.0000000
GO:0045600	positive regulation of fat cell dif	66	0.0451521	1.0000000
GO:0007229	integrin-mediated signaling pathway	119	0.0453845	1.0000000
GO:0070405	ammonium ion binding	94	0.0466047	1.0000000
GO:0035091	phosphatidylinositol binding	292	0.0469776	1.0000000
GO:0006517	protein deglycosylation	43	0.0473682	1.0000000
GO:0002831	regulation of response to biotic st	149	0.0475735	1.0000000
GO:0019731	antibacterial humoral response	55	0.0477479	1.0000000
GO:0031641	regulation of myelination	36	0.0480065	1.0000000
GO:0055006	cardiac cell development	103	0.0483042	1.0000000
GO:0008408	3’-5’ exonuclease activity	93	0.0483098	1.0000000
GO:0006342	chromatin silencing	120	0.0484706	1.0000000
GO:0033003	regulation of mast cell activation	43	0.0485220	1.0000000
GO:0043300	regulation of leukocyte degranulati	45	0.0485545	1.0000000

DMP only KEGG

Gene set min/max limits used were 25/500 (`methylglm()`).

ID	Description	Size	pvalue	padj
04670	Leukocyte transendothelial migration	116	0.0317	1
04380	Osteoclast differentiation	128	0.1265	1
03040	Spliceosome	127	0.1562	1
04530	Tight junction	132	0.1712	1

DMP only Reactome

Gene set min/max limits used were 100/1000 (`methylglm()`).

ID	Description	Size	pvalue	padj
R-HSA-112314	Homo sapiens: Neurotransmitter receptors and postsynaptic signal transmission	156	0.0133	1
R-HSA-72163	Homo sapiens: mRNA Splicing - Major Pathway	179	0.0407	1
R-HSA-72172	Homo sapiens: mRNA Splicing	187	0.0417	1
R-HSA-397014	Homo sapiens: Muscle contraction	206	0.0467	1
R-HSA-112315	Homo sapiens: Transmission across Chemical Synapses	226	0.0590	1
R-HSA-76002	Homo sapiens: Platelet activation, signaling and aggregation	258	0.0671	1
R-HSA-5668914	Homo sapiens: Diseases of metabolism	102	0.0876	1
R-HSA-8957322	Homo sapiens: Metabolism of steroids	147	0.0993	1
R-HSA-156580	Homo sapiens: Phase II - Conjugation of compounds	106	0.1397	1
R-HSA-72203	Homo sapiens: Processing of Capped Intron-Containing Pre-mRNA	236	0.1616	1
R-HSA-109582	Homo sapiens: Hemostasis	602	0.1635	1
R-HSA-2559583	Homo sapiens: Cellular Senescence	186	0.1832	1
R-HSA-166166	Homo sapiens: MyD88-independent TLR4 cascade	100	0.1916	1
R-HSA-937061	Homo sapiens: TRIF(TICAM1)-mediated TLR4 signaling	100	0.1916	1

DMP only

Gene set min/max limits used were 100/500 with a pvalue cut-off of $p < 0.0001$ (`methylgometh()`).

Description	Ont	Size	Count	pvalue	padj	ID
extrinsic component of membrane	CC	284	6	0.0059	1	GO:0019898
cellular response to hypoxia	BP	159	4	0.0071	1	GO:0071456
regulation of cation transmembrane transport	BP	311	6	0.0076	1	GO:1904062
cellular response to decreased oxygen levels	BP	169	4	0.0086	1	GO:0036294
regulation of ion transmembrane transporter activity	BP	234	5	0.0100	1	GO:0032412
regulation of transmembrane transporter activity	BP	241	5	0.0110	1	GO:0022898
regulation of ion transmembrane transport	BP	449	7	0.0118	1	GO:0034765
cellular response to oxygen levels	BP	186	4	0.0123	1	GO:0071453
monovalent inorganic cation transmembrane transporter activity	MF	378	6	0.0126	1	GO:0015077
regulation of transporter activity	BP	256	5	0.0132	1	GO:0032409
regulation of metal ion transport	BP	374	6	0.0142	1	GO:0010959
protein O-linked glycosylation	BP	101	3	0.0165	1	GO:0006493
protein N-terminus binding	MF	106	3	0.0173	1	GO:0047485
response to hypoxia	BP	310	5	0.0191	1	GO:0001666
regulation of potassium ion transport	BP	104	3	0.0202	1	GO:0043266
response to decreased oxygen levels	BP	321	5	0.0214	1	GO:0036293
magnesium ion binding	MF	207	4	0.0230	1	GO:0000287
organelle fusion	BP	117	3	0.0238	1	GO:0048284
voltage-gated ion channel activity	MF	196	4	0.0257	1	GO:0005244
voltage-gated channel activity	MF	196	4	0.0257	1	GO:0022832
response to oxygen levels	BP	344	5	0.0277	1	GO:0070482
cellular potassium ion transport	BP	211	4	0.0280	1	GO:0071804

potassium ion transmembrane transport	BP	211	4	0.0280	1	GO:0071805
potassium channel activity	MF	123	3	0.0291	1	GO:0005267
protein glycosylation	BP	247	4	0.0297	1	GO:0006486
macromolecule glycosylation	BP	247	4	0.0297	1	GO:0043413
ion channel complex	CC	299	5	0.0297	1	GO:0034702
glycosylation	BP	256	4	0.0320	1	GO:0070085
response to endoplasmic reticulum stress	BP	261	4	0.0331	1	GO:0034976
membrane fusion	BP	144	3	0.0332	1	GO:0061025
rRNA processing	BP	203	3	0.0342	1	GO:0006364
symporter activity	MF	142	3	0.0349	1	GO:0015293
UDP-glycosyltransferase activity	MF	145	3	0.0364	1	GO:0008194
transmembrane transporter complex	CC	323	5	0.0370	1	GO:1902495
channel regulator activity	MF	139	3	0.0377	1	GO:0016247
positive regulation of endocytosis	BP	140	3	0.0386	1	GO:0045807
metal ion transmembrane transporter activity	MF	442	6	0.0391	1	GO:0046873
transporter complex	CC	331	5	0.0394	1	GO:1990351
potassium ion transport	BP	237	4	0.0395	1	GO:0006813
ion gated channel activity	MF	339	5	0.0418	1	GO:0022839
response to calcium ion	BP	143	3	0.0442	1	GO:0051592
cation channel complex	CC	217	4	0.0455	1	GO:0034703
gated channel activity	MF	348	5	0.0466	1	GO:0022836
cell maturation	BP	169	3	0.0485	1	GO:0048469
ncRNA processing	BP	368	4	0.0486	1	GO:0034470
voltage-gated cation channel activity	MF	141	3	0.0516	1	GO:0022843
platelet activation	BP	151	3	0.0528	1	GO:0030168
anatomical structure maturation	BP	154	3	0.0560	1	GO:0071695

potassium ion transmembrane transporter activity	MF	159	3	0.0571	1	GO:0015079
rRNA metabolic process	BP	238	3	0.0589	1	GO:0016072
regulation of cation channel activity	BP	160	3	0.0595	1	GO:2001257
response to ketone	BP	190	3	0.0625	1	GO:1901654
transferase activity, transferring hexosyl groups	MF	204	3	0.0635	1	GO:0016758
proximal promoter DNA-binding transcription repressor activity, RNA polymerase II-specific	MF	169	3	0.0638	1	GO:0001078
antigen receptor-mediated signaling pathway	BP	183	3	0.0686	1	GO:0050851
condensed chromosome kinetochore	CC	103	2	0.0721	1	GO:0000777
ribosome biogenesis	BP	277	3	0.0728	1	GO:0042254
glycoprotein biosynthetic process	BP	323	4	0.0728	1	GO:0009101
extrinsic component of plasma membrane	CC	161	3	0.0751	1	GO:0019897
cytoplasmic side of membrane	CC	171	3	0.0813	1	GO:0098562
vesicle budding from membrane	BP	100	2	0.0825	1	GO:0006900
ion channel activity	MF	423	5	0.0829	1	GO:0005216
blood coagulation	BP	327	4	0.0830	1	GO:0007596
coagulation	BP	333	4	0.0847	1	GO:0050817
hemostasis	BP	332	4	0.0871	1	GO:0007599
substrate-specific channel activity	MF	438	5	0.0872	1	GO:0022838
condensed chromosome, centromeric region	CC	116	2	0.0877	1	GO:0000779
pattern specification process	BP	432	5	0.0923	1	GO:0007389
channel activity	MF	462	5	0.0959	1	GO:0015267
passive transmembrane transporter activity	MF	463	5	0.0963	1	GO:0022803
segmentation	BP	102	2	0.0965	1	GO:0035282
regulation of body fluid levels	BP	484	5	0.0969	1	GO:0050878
