# Supplementary material for: DNA methylation associated with postpartum depressive symptoms overlaps findings from a genome-wide association meta-analysis of depression
Source: Clin Epigenetics. 2019 Nov 28;11:169. doi: 10.1186/s13148-019-0769-z (PMC6883636; doi:10.1186/s13148-019-0769-z)
Supplement: Supplementary file 3 — Additional file 3. Enrichment testing of Psychiatric Genomic Consortium (PGC) supplemental methods. This file contains additional details about how the 95% confidence intervals were calculated for the PGC enrichment analysis. [file 13148_2019_769_MOESM3_ESM.pdf]

**Enrichment testing for overlap with Psychiatric Genomics Consortium (PGC) genome-wide association study meta-analysis of depression**

In order to calculate the 95% confidence interval for the number of observed overlaps between the differentially methylated regions in this study and the PGC, bootstrapping (k=1000 permutations) was used. The frequency that overlaps of 0-10 PGC loci occurred during the permutation analysis can be found in Table S1.

Table S1: Results of bootstrapping confidence intervals for overlap with the PGC GWAS meta-analysis of depression

| Overlaps | 0 | 1 | 2 | 3 | 4 | 5 | 6 | 7 | 8 | 9 | 10 |
|---|---|---|---|---|---|---|---|---|---|---|---|
| Frequency | 57 | 138 | 225 | 228 | 165 | 90 | 58 | 29 | 7 | 1 | 2 |

Frequency = the number of times each overlap occurred in 1000 permutations.
PGC = Psychiatric Genomics Consortium; GWAS = genome-wide association study

Table S2: Overlap of Significant Differentially Methylated Regions and PGC GWAS meta-analysis of depression

| Chromosome | DNAm.start | DNAm.end | PGC.start | PGC.end |
|---|---|---|---|---|
| chr5 | 88178977 | 88180332 | 87443000 | 88244000 |
| chr6 | 30028447 | 30029721 | 27738000 | 32848000 |
| chr16 | 72127419 | 72128393 | 71631000 | 72849000 |

Genomic annotations used hg19 build 37.
