# Supplementary material for: DNA methylation associated with postpartum depressive symptoms overlaps findings from a genome-wide association meta-analysis of depression
Source: Clin Epigenetics. 2019 Nov 28;11:169. doi: 10.1186/s13148-019-0769-z (PMC6883636; doi:10.1186/s13148-019-0769-z)
Supplement: Supplementary file 4 — Additional file 4: Supplement to Figure 2. This file contains two additional versions of the differentially methylated region (DMR) highlighted in Fig. 2. The top figure shows the ComBat-adjusted methyl values for each participant colored by self-identified Census-based race category. The points have been jittered left/right to ease visualization by reducing over-plotting. No vertical adjustment was made. The bottom figure shows the mean methyl values for each probe contained in the DMR by self-identified Census-based race category. [file 13148_2019_769_MOESM4_ESM.pdf]

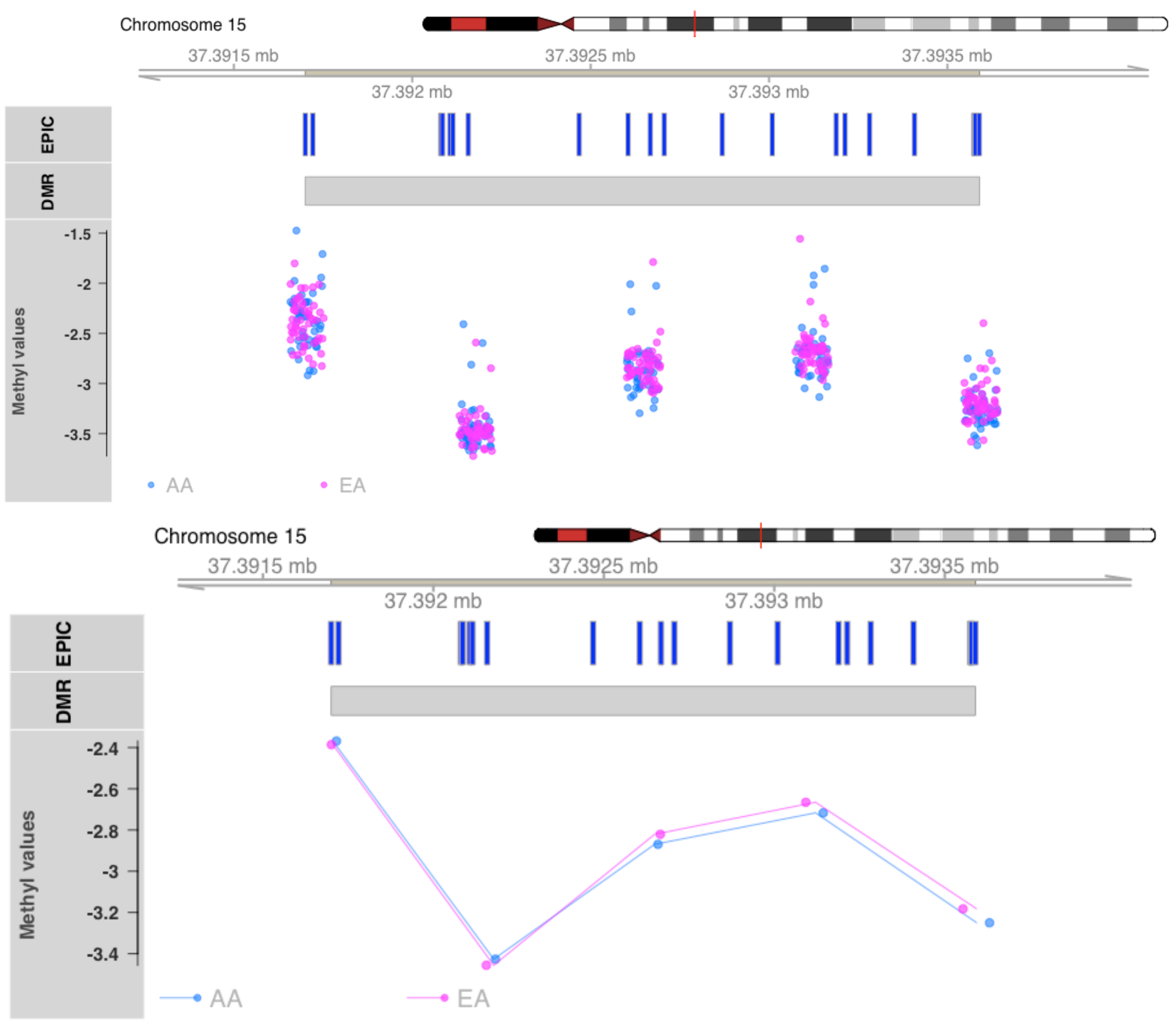

Figure S1.

Figure S1. Supplement to Figure 2. In the bottom figure, the ComBat-adjusted methyl values for each participant are shown for each of the five probes used to build the differentially methylated region on chromosome 15. The color indicates self-reported Census-based race category (blue = African-American; red = European-American). The methyl values have been jittered left/right to reduce over plotting, but the height (i.e., methyl value) has not been altered. Mean-level methyl values for AA and EA participants are shown for each probe.
