# Supplementary material for: DNA methylation associated with postpartum depressive symptoms overlaps findings from a genome-wide association meta-analysis of depression
Source: Clin Epigenetics. 2019 Nov 28;11:169. doi: 10.1186/s13148-019-0769-z (PMC6883636; doi:10.1186/s13148-019-0769-z)
Supplement: Supplementary file 5 — Additional file 5. Additional Figures. This file contains four figures: quantile-quantile plots for the DMP and DMR analyses and histograms of the distribution of when PREG postpartum study visits occurred (i.e., time since birth) and the distribution of EPDS total scores by self-reported lifetime history of MD (assessed using an extended self-report version of the CIDI-SF). [file 13148_2019_769_MOESM5_ESM.pdf]

Supplemental Figures

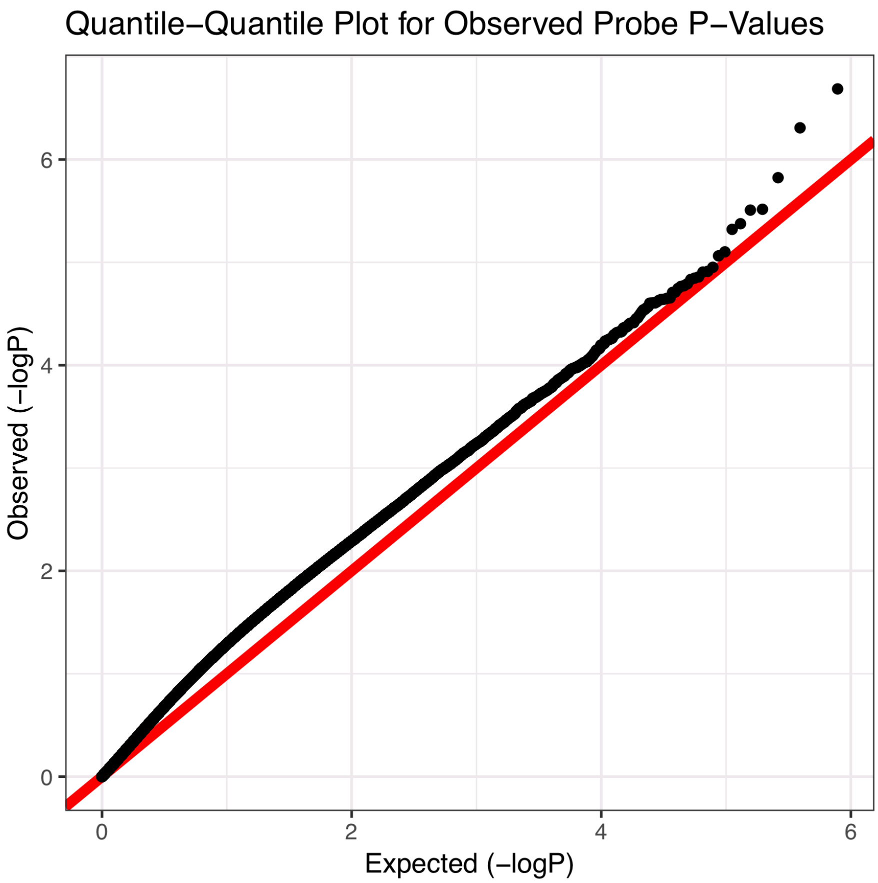

Figure S1: Quantile-Quantile Plot for Individual Site Analysis. The red line indicates the expected p values for a null association.

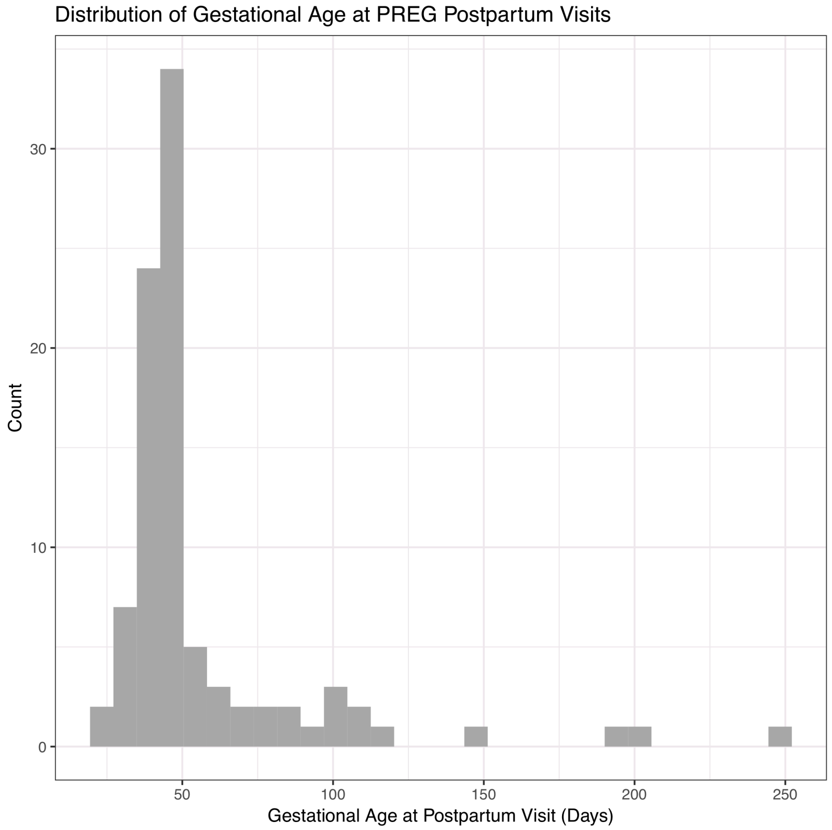

Figure S2: Distribution of Gestational Age at PREG Postpartum Study Visits

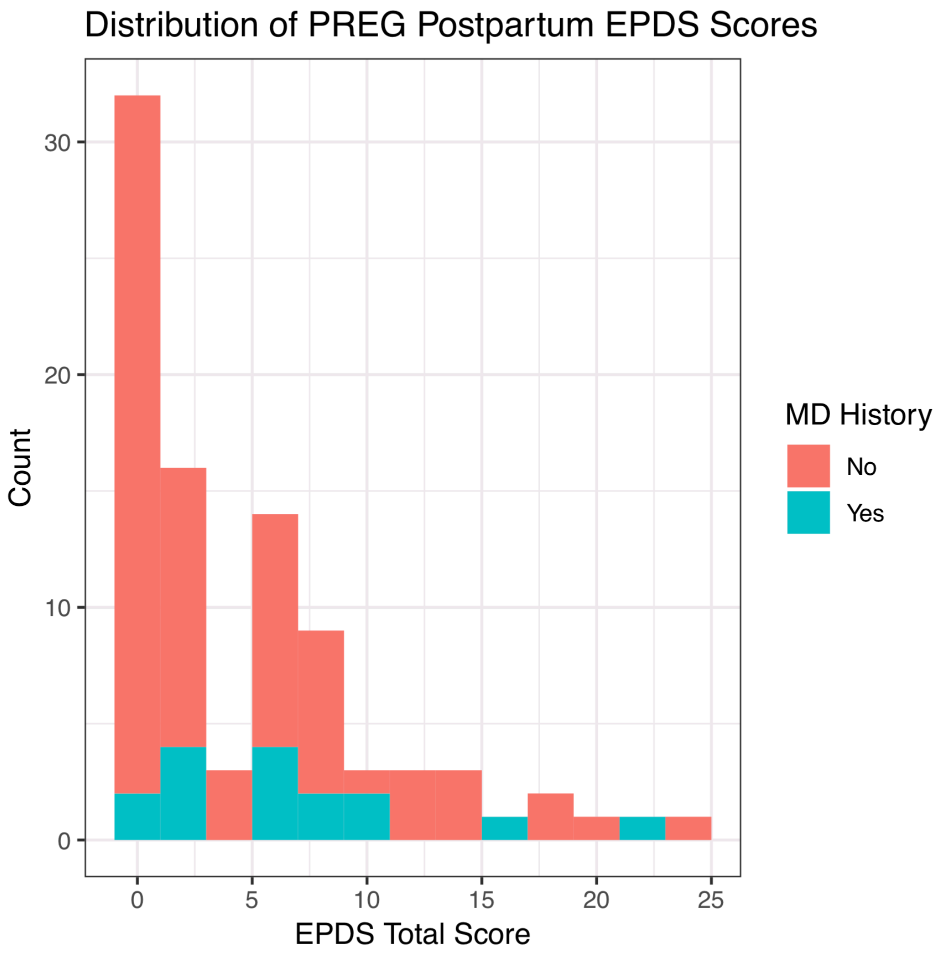

Figure S3: Distribution of PREG Postpartum EPDS Scores. This figure shows the distribution of PREG postpartum Edinburgh Postnatal Depression Scale total scores by lifetime history of major depression (MD) for the participants (n=89) with valid responses to the MD diagnostic items in the extended self-report version of the Composite International Diagnostic Interview-Short Form.

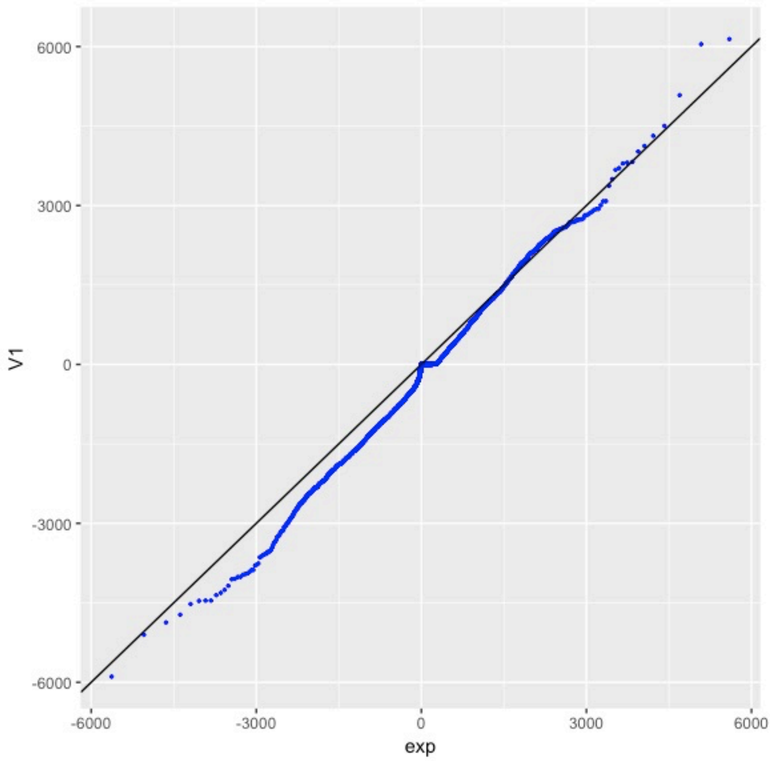

Figure S4: Quantile-Quantile Plot of Genomic Regions. Each point represents one region, including regions of size 1 CpG site. The units on the x and y axes represent the area under the curve (AUC) of a region. The expected and observed AUC values are shown on the x and y axes, respectively. AUC was calculated using the trapezoidal rule where AUC equals length times height. Length is the genomic distance between CpG sites, and height is the test statistics such that stronger associations have taller lengths. Thus, all single CpG site regions have an AUC value of zero. Significant differentially methylated regions were identified using the Significance Analysis of Microarrays, which performs row-wise comparisons across all permutation sets, and calculates a false discovery rate (FDR). To prevent strong outliers from inflating the FDR, the two regions with the most extreme AUC values were excluded from the FDR calculation.
